# 

**Published:** 1906-05-19

**Authors:** 


					the hospital. BUI f J May 19th 1906.
TALr
Mirtca naspt.taa
No. l,025y Vol. XL. KSBSSJfl Saturday,
May 19th, 1906.
rbr&Tm"] PRICE THREEPENCE

				

